# A Mental Imagery Micro-Intervention to Increase Positive Affect in Outpatient CBT Sessions (PACIfIC): Study Protocol of a Randomized Controlled Implementation Trial

**DOI:** 10.32872/cpe.7043

**Published:** 2022-06-30

**Authors:** Jan Schürmann-Vengels, Philipp Pascal Victor, Patrizia Odyniec, Christoph Flückiger, Tobias Teismann, Ulrike Willutzki

**Affiliations:** 1 Department of Psychology and Psychotherapy, Universität Witten/Herdecke, Witten, Germany; 2 Department of Clinical Psychology and Psychotherapy, University of Zurich, Zurich, Switzerland; 3 Mental Health Research and Treatment Center, Ruhr-Universität Bochum, Bochum, Germany; Philipps-University of Marburg, Marburg, Germany

**Keywords:** positive affect, mental imagery, psychotherapy process, cognitive behavioral therapy, randomized controlled trial, multilevel models

## Abstract

**Background:**

Recent findings indicated that mental disorders are associated with both an up-regulation of negative affect and a down-regulation of positive affect (PA) as distinct processes. Established treatment approaches focus on the modification of problems and negative affect only. Experimental paradigms in healthy samples and research on strengths-based approaches showed that fostering PA may improve psychotherapy process and outcome. Specific and easily implementable interventions targeting PA in treatment sessions are scarce. Mental imagery was shown to be a promising strategy for boosting positive emotional experiences.

**Method:**

The PACIfIC-study is planned as a longitudinal randomized-controlled trial in the context of cognitive behavioral therapy, implemented at a German outpatient training and research center. In the process analysis, trajectories of PA over the first twelve treatment sessions will be examined with weekly questionnaires. In the intervention analysis, a six-minute positive mental imagery intervention to enhance PA will be developed and tested. The intervention is implemented with loudspeakers at the beginning of each session for a standardized induction of PA. The experimental group will be compared to an active control group (neutral mental imagery) and treatment as usual. Procedures in all treatment arms are parallelized. Main outcomes after twelve sessions of psychotherapy will be psychosocial resources, resilience and self-esteem (theory-driven), as well as psychopathology and working alliance (secondary outcome). Multilevel modeling will be conducted to address the nested data structure.

**Conclusion:**

Study results may have implications on the consideration of positive constructs in mental disorders and the implementation of strengths-based interventions in psychotherapy.

## Highlights

- This planned study will examine the trajectories of positive and negative affect during early CBT.
- Development and pilot-test of a six-minute positive mental imagery intervention are described.
- Possible effects of the positive mental imagery implementation of treatment outcome are discussed.

Treatments like cognitive behavioral therapy (CBT) have shown effectiveness for various mental disorders (e.g. Hofmann et al., 2012). However, there is a lack of knowledge about basic processes in mental disorders and psychotherapy. Affect dysregulation is recently discussed as a factor for the maintenance of psychopathology. Affect is defined as the subjective experience of an emotional state and is differentiated by its valence (Hofmann, 2016). Positive and negative affect are assumed to be correlated, but separate constructs (Larsen et al., 2017; Watson et al., 1999). Dysfunctional up-regulation of negative affect (NA) is a common feature in mental disorders (Aldao et al., 2010) and is successfully modified by CBT (Boumparis et al., 2016; Sauer-Zavala et al., 2012). In contrast, the impact of positive affect (PA) in psychopathology and psychotherapy is not well established. Regarding this research gap, we designed the Positive AffeCt and mental Imagery In the process of Cognitive behavioral therapy (*PACIfIC)*-study.

## PA and Psychological Processes

PA is characterized by various emotions and moods with a subjective pleasant valence. According to the broaden-and-build theory of positive emotions, PA initiates a multistage upward spiral process (Fredrickson, 2001; Garland et al., 2010). In particular, research findings from healthy samples showed that PA leads to a broadening of attention as well as thought and action repertoires. Positive mood inductions in experimental paradigms increased visuospatial attention as well as information processing (Phaf, 2015; Pourtois et al., 2017; Vanlessen et al., 2016). High PA was related to better performance in category building and creativity tasks (Baas et al., 2008; Nadler et al., 2010). Observational and experimental studies showed that such broadening, in turn, was associated with a reciprocal increase of psychosocial resources, resilience and mental health (Garland et al., 2010; Griffith et al., 2021). The broaden-and-build theory, therefore, hypothesized that an increase in PA will lead to higher levels in these specific variables. Hence they may help to evaluate interventions that target an increase of PA. Brief descriptions of these constructs are presented in the following: Psychosocial resources were defined as positive and functional aspects of a person or his/her environment (e.g. optimism, social support; Taylor & Broffman, 2011). Previous findings indicated moderate to high correlations between different constructs and the possibility to assess a generic perception of inherent resources (Goldbach et al., 2020; Taylor & Broffman, 2011; Victor et al., 2019). Psychological resilience was defined as the potential to successfully adapt to adversities and stressors (Davydov et al., 2010). Previous studies found that resilience is a dynamic trait, which is both influenced by internal and external experiences and changeable by purposeful interventions (Connor & Davidson, 2003; Mealer et al., 2014). Self-esteem is an important part of mental health and was defined as the degree, a person positively consider his/her characteristics or abilities (Brown, 2007). Baseline parameters and trajectories of self-esteem and PA were strongly related in observational and intervention studies (Garland et al., 2010; Wood et al., 2003). Most concepts have focused on a global evaluation of self-esteem rather on specific facets (Rosenberg et al., 1995).

## PA in Psychopathology and Psychotherapy

Carl et al. (2013) pointed out that the down-regulation or dampening of PA is an independent process in mental disorders. In a prospective paradigm, baseline anxiety and depressive symptoms were related to lower rates of daily positive emotional reactivity and decreased levels of PA in the subsequent 14-day period (Carl et al., 2014). Specific analyses found decreased levels of PA for various mental disorders (Cohen et al., 2017; Eisner et al., 2009; Thompson et al., 2016). These findings support the idea to consider and foster PA by psychotherapeutic interventions. Concurrently, classical CBT treatments had only small effects on PA in patients with major depression, *g* = 0.41, *p* = .001, and anxiety disorders, *g* = 0.37, *p* = .001 (Boumparis et al., 2016; Wilner Tirpak et al., 2019).

An established approach to foster positive constructs in psychological treatments is resource activation. The activation of patients’ strengths and resources is regarded as a change mechanism in psychotherapy and was significantly associated with patients’ PA and treatment outcome in CBT sessions (Flückiger et al., 2009; Mander et al., 2013). Observer ratings showed that successful CBT sessions were characterized by higher levels of resource activation and PA, particularly at the beginning of treatment sessions (Gassmann & Grawe, 2006; Smith & Grawe, 2003). Moreover, Chui et al. (2016) found that higher initial PA of patients in psychotherapy sessions lead to both more PA of therapists and better rated post-session collaboration. Although these studies indicate the feasibility to increase PA in psychotherapeutic settings and its promotive influences on symptom improvement and working alliance, economical strategies that directly targeting PA are lacking.

## Mental Imagery as a Strategy to Increase PA

Mental imagery is defined as “representations and the accompanying experience of sensory information without a direct external stimulus” (Pearson et al., 2015, p. 590). Compared to other interventions, mental imagery was found to be more effective in evoking emotions (Holmes et al., 2009; Holmes & Matthews, 2010; Schubert et al., 2020). Recent research approaches compared the imagination of positive versus neutral contents to differentiate its affective impact (Grol et al., 2017). In clinical settings, most studies of positive mental imagery were conducted as single interventions to promote PA in patients with major depression: these trainings were associated with reduced depressive symptoms and anhedonia, as well as increased optimism, positive self-referent cognitions and behavioral activation in clinical samples (Blackwell et al., 2015; Dainer-Best et al., 2018; Ji et al., 2017; Renner et al., 2017). Alternatively, positive mental imagery has also been discussed to enhance anxiety (Wallace & Alden, 1997) and trigger dissociation (Brewin et al., 2010). Another analysis by O’Donnell et al. (2017) showed that positive mental imagery training in individuals with high hypomanic experiences led to a dysfunctional amplification of positive mood. Thus, while there is substantial evidence that fostering PA may be a promising intervention strategy also in psychotherapy, we do not know whether the systematic implementation of positive mental imagery has a beneficial impact on psychotherapy process and outcome.

Specific analyses found important factors influencing the promotion of PA in mental imagery: practicing mental imagery repeatedly (Blackwell et al., 2015; O’Donnell et al., 2017), including various sensory modalities (Holmes et al., 2008), imaging personally relevant situations, aspects or perspectives (Quoidbach et al., 2009) and employing a field perspective (Grol et al., 2017).

## Current Study

The dysregulation of PA is a prominent and distinct factor in psychopathology and should be focused in CBT. Higher experience of PA may broaden patients’ receptivity in treatment sessions and may enable goal-related approaching behaviors. However, there is a massive lack of knowledge about PA in the therapeutic process. To the best of our knowledge, no study has attempted to activate PA via an economical intervention in CBT sessions.

Within the *PACIfIC*-study, both a process and an intervention analysis will be conducted. In the process analysis, we will examine the course of PA and NA in the first twelve sessions of CBT treatments. Therefore, primary outcome in the process analysis will be the slope of PA and NA. Further measures of resource activation, working alliance and psychopathology after each of the twelve sessions will be included in the process analysis to analyze their relation to PA (within and between sessions).

In the intervention analysis, we will examine the effects of a six-minute positive mental imagery intervention during an early phase of psychotherapy. The aim of this micro-intervention is to foster patients’ in-session PA, which may lead to increased levels of subjective resources, resilience, and self-esteem (theory-driven outcome) as well as improvements in psychopathology and working alliance (secondary outcome). Changes in the theory-driven outcome variables are expected due to the specific effects of increased PA according to the broaden-and-build theory of positive emotions (Fredrickson, 2001). Changes in the secondary outcome variables are expected due to found effects of resource activation and shared positive emotions in treatment (Chui et al., 2016; Flückiger et al., 2009). Patients will be randomized into one of three parallel treatment arms with a 1:1:1 allocation: CBT + positive mental imagery micro-intervention (PMI), CBT + neutral mental micro-intervention (NMI), or treatment as usual (TAU). Two active mental imagery micro-interventions are planned to differentiate the specific effect of a PA induction within treatment sessions. The study serves the following objectives:

1. To explore the trajectories of PA and NA in an early phase of CBT treatment.
2. To develop and test the feasibility of a brief intervention to promote PA in psychotherapy sessions.
3. To analyze the impact of this intervention on the therapeutic process between and within CBT sessions and intermediate outcomes.

We hypothesize that PA will increase, while NA will decrease during the first 12 sessions of therapy. According to the specific effects postulated by the broaden-and-build theory of positive emotions, we further hypothesize that patients in the PMI will show higher in-session PA and higher levels of subjective resources, resilience, and self-esteem compared to the other conditions.

## Method

### Design

**Figure 1:**
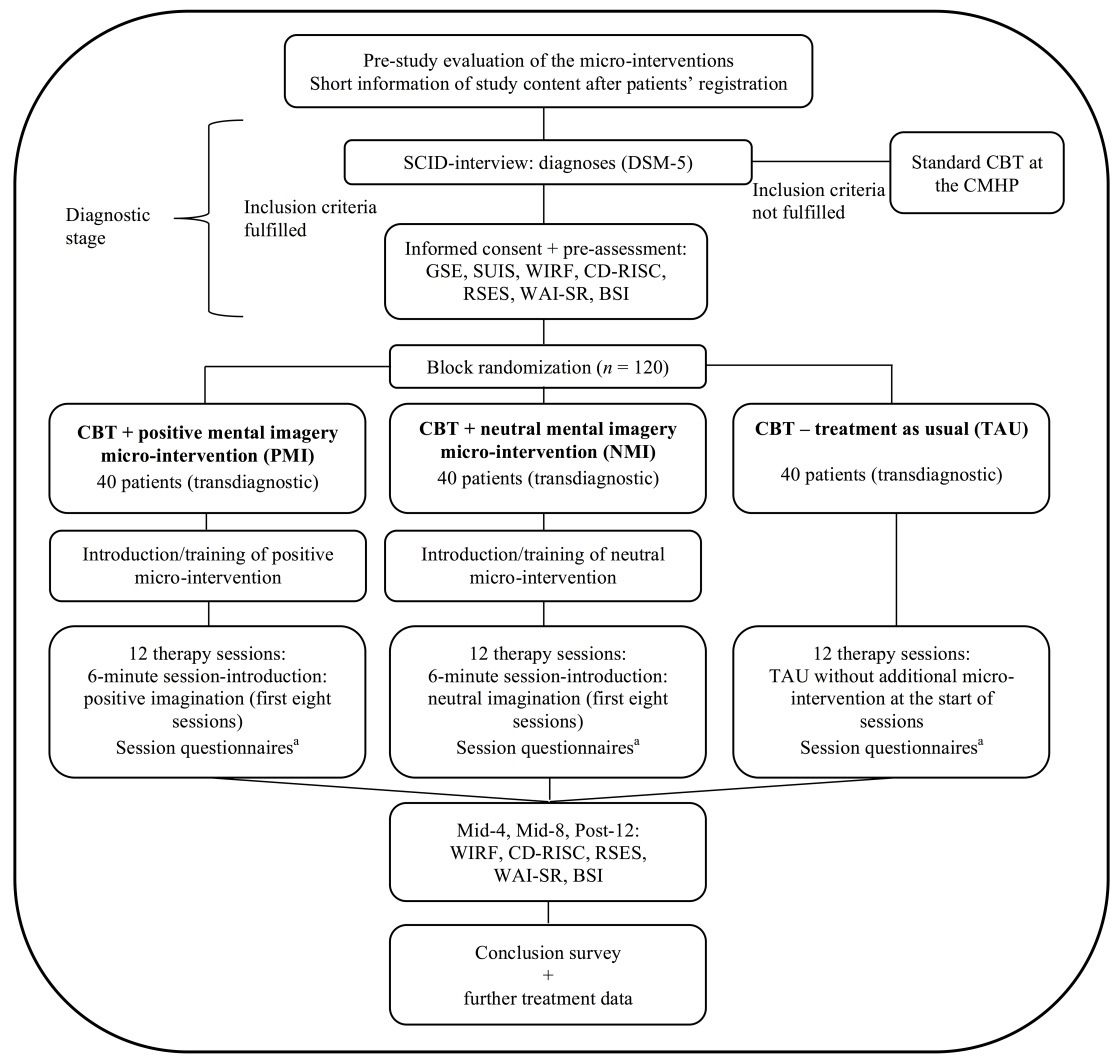
Flowchart of Study Design *Note*. a = session questionnaires: Positive and Negative Affect Schedule (PANAS), Single-item mood scaling, Multiperspective Assessment of Change Mechanisms in Psychotherapy (SACiP-RA), Working Alliance Inventory – Short Revised (WAI-SR), Short version of Derogatis Symptom Checklist (SCL-K-9); BSI = Brief Symptom Inventory; CD-RISC = Connor-Davidson Resilience Scale; CMHP = Center of Mental Health and Psychotherapy; GSE = General Self-Efficacy Scale; RSES = Rosenberg Self-Esteem Scale; SCID = Structured Clinical Interview according to DSM-5 criteria; SUIS = Spontaneous Use of Imagery Scale; WAI-SR = Working Alliance Inventory – Short Revised; WIRF = Witten Strengths and Resource Form.

Figure 1 displays a SPIRIT chart of the planned study design. The study is a randomized controlled implementation trial with three parallel treatment arms using a 1:1:1 allocation ratio. A blocked randomization with blocks of variable length conducted by a random-number generator (random.org) will be performed. Block length will be determined randomly (9, 12 or 15 units), before conditions will be randomized within all blocks separately. An independent research assistant will develop the block list and conduct the randomization. Patients will be randomized to one of the following arms: CBT + positive mental imagery induction (PMI), CBT + neutral mental imagery induction (NMI) and TAU. All arms include an individual CBT treatment. A cross-therapist design in which any therapist can deliver all three conditions is applied. Randomization will be focused on patients only, so that therapists will not see a fixed number of patients per condition. However, we expect that the block randomization will enable an approximately equal number of patients in all conditions per therapist. All participants are blind to the conditions and specific hypotheses. According to the CONSORT statement concerning the criteria of a pragmatic randomized trial (Zwarenstein et al., 2008) therapists and study coworkers conducting the information meetings are not blind to allocation. Researchers involved in data collection and evaluation will be blind to condition labels. An independent researcher will analyze study data with non-identifying codes of the conditions. The study includes a longitudinal design with an initial diagnostic phase (four to five sessions) and the following twelve psychotherapy sessions. Outcome for the process analysis will be gathered directly after each treatment sessions. In the intervention analysis, assessments will be made every forth session: at the start of treatment (pre), after fourth (mid-4), after eighth (mid-8) and after twelfth treatment session (post-12). In addition, videotapes of treatment sessions 2, 5 and 8 will be analyzed with an observer rating.

### Participants

#### Patients

A total of 120 patients will be recruited at the Center of Mental Health and Psychotherapy (CMHP), an outpatient training and research center for CBT at Witten/Herdecke University, Germany. General inclusion criteria will be as follows: (1) psychotherapy outpatient, (2) at least one mental disorder according to DSM-5 criteria, (3) at least 18 years of age. General exclusion criteria will be as follows: (1) current diagnosis of a severe episode of major depressive disorder, (2) suffering from a psychotic disorder, (3) suffering from substance use disorder, (4) current episode of (hypo)mania, (5) current suicidal risk, (6) extensive experiences with guided mental imagery interventions (two or more interventions in prior treatment settings), (7) insufficient German language skills, (8) currently receiving another psychological treatment. Prescribed medications for anxiety or depressive disorders do not lead to exclusion from the study. The presence of comorbidities does not result in exclusion from the study.

A power analysis with G*Power (Faul et al., 2007) based on effect sizes from relevant studies (Flückiger et al., 2016; Willutzki et al., 2004) was conducted to determine sample size. The detection of a small to moderate effect (Cohen’s *f* = 0.15) for the interaction between time (pre, mid-4, mid-8, post-12) and treatment condition (PMI vs. NMI vs. TAU) [mixed model analysis of variance (ANOVA), within-between-interaction, α = .05, power = .80, number of groups = 3, number of measurements = 4, pre-post correlation = .50, non-sphericity correction = 1] resulted in a sample size of 78 patients. Considering possible dropouts, we will recruit up to 120 patients. Power analysis of a repeated measurement ANOVA is comparable to multilevel models (MLM; Baldwin et al., 2014).

#### Therapists

20-25 therapists will be recruited at the CMHP. All therapists have at least a master’s degree in psychology. Both, licensed CBT therapists and therapists in advanced CBT training will take part in the study. Trainee therapists have at least one year of clinical experience before they start treatments in the CMHP. Parallel to the study, trainee therapists take part in 600 hours of practice-based workshops as a part of CBT training protocols in Germany. Therapists participate in 90-minute supervision in small groups on a weekly basis (general clinical supervision, not study-specific). Every therapist in the CMHP will be informed about study procedures in small group meetings of approx. 30 minutes conducted by JSV.

### Standard Procedure at CMHP

Adult patients with various types of mental disorders receive treatment by approximately 20 licensed CBT therapists resp. trainee therapists. The CMHP has eight rooms for providing psychotherapy. All rooms are fully equipped with video and audio recording. Computer-assisted psychometric assessments during therapy are standard procedures at the CMHP and regularly reviewed by staff members. Personal and treatment-specific data is managed with a software called AmbOS. To promote data quality, fixed schedules of assessments for patients and therapists were included. Patients interested in a CBT treatment have a first phone contact and a one-session consultation with a licensed therapist. They are then listed on an internal waiting list (with currently on average six months waiting time). Patients are contacted by a therapist and invited to a standardized diagnostic phase. The diagnostic phase includes four to five sessions with the following order: exploration and treatment consent, Structured Clinical Interview for DSM-5 Disorders (SCID; Beesdo-Baum et al., 2019), biographic work, situation analysis. After the diagnostic phase, a CBT treatment according to the German health system is offered.

### Development of the Micro-Interventions

A systematic literature search of interventions to foster PA was conducted, indicating positive mental imagery as a promising strategy. Important aspects to boost emotional experiences in imagery interventions were identified based on relevant studies (e.g. Grol et al., 2017; Holmes et al., 2008). Procedures of positive and neutral interventions used in these studies are screened in detail. Based on this information, we developed a first version of the PMI in a six-minute format. Next, eleven therapists piloted-tested the intervention with 25 different patients regarding its practical implication. Therapists conducted the intervention within treatment sessions. An anonymous survey was conducted, in which patients and therapists described positive and critical aspects of the intervention independently. We reformulated the intervention script, based on this feasibility information, to its final version. The NMI script was parallelized. Scripts for both interventions can be found in the Appendix (see Supplementary Materials). We decided to record the interventions on audiotapes that will be played at the start of each treatment session to increase standardization (inspired by the PrOMET-study by Mander et al., 2019). Both interventions are spoken and recorded by UW. The audiotapes will be played on bluetooth speakers (Anker SoundCore Mini). Two loudspeakers (grey: PMI; black: NMI) are installed in every therapy room.

### Conditions and Experimental Session

All three conditions will be parallelized and include a CBT treatment based on an individual case conception. Every session will start with an initial greeting of patients and the start of video recording.

Experimental sessions will be conducted from session one to eight in the active conditions. In each experimental session of the PMI condition, a grey loudspeaker will be placed on a table in front of patient and therapist with on average one-meter distance. The therapist will carry out the mood scaling by asking the patient to rate his/her mood in the present moment from one (very bad mood) to ten (very good mood). After that, therapists will start the record of the PMI (duration about six minutes). Patients are guided to imagine a positive situation from the last week. Directly after the micro-intervention, the same mood scaling will be conducted again. At the end of the intervention, patients are instructed to communicate the content of their imagination with their therapist for about one minute. After completion, the regular CBT session will begin. After each session patients will complete session questionnaires. Procedures of the experimental sessions in the NMI condition will be parallelized to the description above. At session start, the micro-intervention will be performed with a black speaker with the instruction to imagine a non-emotional situation within the last week. In the control condition, standard CBT will be conducted without additional micro-intervention.

### Study Course From Patients’ Perspective

**Figure 2:**
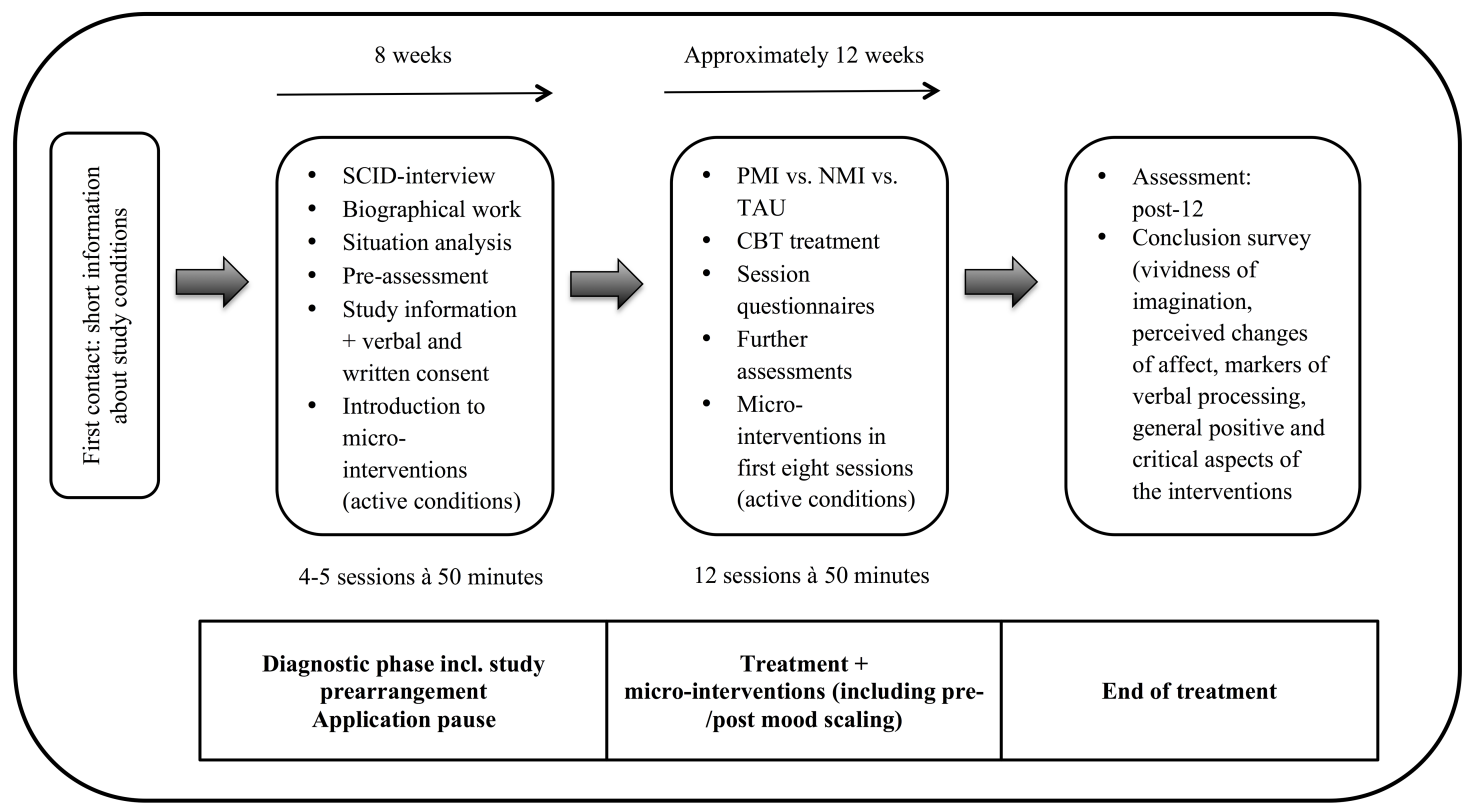
Study Course From Patients’ Perspective

Figure 2 shows the study course from patients’ perspective. Patients will get a short-information about the studies objectives and procedures within in the first session of the diagnostic phase. If interested, a study coworker will contact them for an additional meeting. Patients will receive written and verbal study information in this meeting and will sign informed consent. It will be emphasized that study participation is voluntary with the option to revoke study participation at any time without reasons and/or disadvantages. Patients in both active conditions will receive an introduction to the respective mental imagery intervention, including cooperative exploration of specific contents (situation imagery as detailed as possible, sensory modalities, field perspective), examples of positive/neutral situation in the their life, and a practice of the respective intervention. Furthermore, possible difficulties with the interventions will be discussed. The study coworkers will educate patients how to handle it if they fall out of their imagination during the intervention. After the diagnostic phase, all patients will run through twelve CBT sessions. Patients will receive session questionnaires directly after each session. Additional measurements after every forth session will be included in the study.

### Measures

Table 1 provides an overview of all measures and their application in the study.

**Table 1:** Application Plan of Measures

Measures	Pre	Session by Session	Measurement waves		
			Mid-4	Mid-8	Post-12
Session questionnaires					
Mood scaling		1-12			
Positive and Negative Affect Schedule (PANAS)	x	1-12			
Resource activation (SACiP-RA)	x	1-12			
Working-Alliance-Inventory (WAI-SR)	x	1-12			
Short version of Symptom-Checklist (SCL-9-K)	x	1-12			
Clinical assessment					
Witten Strengths and Resource Form (WIRF)	x		x	x	x
Connor-Davidson Resilience Scale (CD-RISC)	x		x	x	x
Rosenberg Self-Esteem Scale (RSES)	x		x	x	x
Brief Symptom Inventory (BSI)	x		x	x	x
General Self-Efficacy Scale (GSE)	x				
Spontaneous Use of Imagery Scale (SUIS)	x				
Observer Rating					
Resource-oriented micro-process analysis (ROMA)		2nd, 5th, 8th session			

*Note*. pre = baseline scores; mid-4 = assessment after fourth sessions; mid-8 = assessment after eighth session; post-12 = final assessment after twelfth session.

#### Process Analysis – Primary Outcome

##### Session Questionnaire I

To assess PA and NA of patients, we will apply the Positive and Negative Affect Schedule (PANAS; Krohne et al., 1996). The PANAS is an internationally used 20 item self-report. As described, affect is defined as the subjective experience of an emotional state and is mostly differentiated by positive versus negative valence (Hofmann, 2016). Participants will be asked to rate the items according to how they feel "in the current moment". Two subscales of global PA (ten items, range: 1-5) and global NA (10 items, range: 1-5) will be used. Item examples are shown in the following: “Indicate the extent you feel this way in the current moment - Proud (global PA); - Nervous (global NA). Both scales have shown good internal consistency (PA: α = .85, NA: α = .86) and are widely validated (Krohne et al., 1996).

#### Process Analysis – Further Measures

##### Session Questionnaire II

Mood scaling – a single-item mood scaling (“How do you feel in the present moment?”) will be used as an economical assessment of affect. Patients will be asked to rate their mood on a scale from one (very bad mood) to ten (very good mood). Various short measures of mood were applied in previous studies: These instruments showed practicability and content validity (high correlation with measures of depressive mood) in clinical samples, especially for visual mood scales (Ahearn, 1997; Luria, 1975). Moreover, Van Rijsbergen et al. (2014) showed the transferability of these results for a verbal single-item mood rating. The mood scaling will be used: (1) as a session questionnaire directly after each of the twelve sessions; (2) as an evaluation instrument of the mental imagery micro-interventions in the active conditions (mood scaling before and after the micro-intervention, see conditions and experimental sessions). We decided to include a further affect measure, additionally to the PANAS, because of the simple use of the single item mood scaling in therapy sessions. It is also included as a session questionnaire to analyze the post-hoc correlation between the mood scaling and the PANAS to check construct validity of the single-item measure.

##### Session Questionnaire III

To assess levels of resource activation from patients’ perspective, we will apply the subscale resource activation of the Scale for the Multiperspective Assessment of Change Mechanisms in Psychotherapy (SACiP-RA; Mander et al., 2013). Resource activation refers to transdiagnostic change processes in therapy where strengths or potentials of the patient become perceptible and are used in treatment sessions (Grawe & Grawe-Gerber, 1999). The instrument was included based on prior findings, indicating the association of PA and in-session resource activation (e.g. Flückiger et al., 2009). The subscale consists of three items (range: 0-4). Items of the SACiP-RA were developed based on the Bern Post Session Questionnaire, an established therapy process measure (Flückiger et al., 2010). Item example: “In today’s session, I felt where my strengths lie.” The subscale has displayed good internal consistency (α = .71) and significant associations with treatment outcome (Mander et al., 2013).

##### Session Questionnaire IV

To assess quality of the therapeutic alliance, we will apply the Working Alliance Inventory – Short Revised (WAI-SR; Wilmers et al., 2008). We decided to include the WAI-SR as a process measure to analyze its association with PA within and between sessions. Based on prior studies, we expect that patients report better alliance directly after sessions with high levels of PA (Chui et al., 2016). Furthermore, we want to analyze whether PA and the working alliance will develop parallel in the process of treatment. The WAI-SR is an internationally used 12 items self-report of therapeutic alliance measuring bond, goals and tasks in psychotherapy based on feedback of patients concerning the current therapy session. Items are answered on a likert scale from one to five. Item example: “My therapist and I respect each other.” The WAI-SR is considered the gold standard in alliance assessment with excellent psychometric properties and outcome prediction (Horvath et al., 2011).

##### Session Questionnaire V

To assess general psychopathology, we will apply the short version of Derogatis Symptom Checklist (SCL-K-9; Klaghofer & Brähler, 2001). We decided to include the SCL-K-9 as a process measure to analyze its association with PA within and between sessions (e.g. parallel development of increase of PA and improvement in symptoms). The short version with nine items (range: 0-4) is an internationally used self-report. Item example: “During the last past seven days, how much were you distressed by: finding it difficult to start something.” The short version has shown good internal consistency (α = .87) as well as high correlations to the original version (Petrowski et al., 2019).

#### Intervention Analysis – Primary Outcome

##### Subjective Resources of Patients

Witten Strengths and Resource Form (WIRF; Victor et al., 2019). As described, psychosocial resources were defined as positive and functional aspects of a person or his/her environment (Taylor & Broffman, 2011). The instrument assesses a generic perception of resources rather than separate positive aspects. Therefore, it measures the internal evaluation of a person’s inherent resources. This subjective perception should be differentiated to the therapeutic process of resource activation. The WIRF is a self-report with 37 items (range 0-5). In our study we will use a total score of resources in the context of current problems (12 items). Item example: “I am dealing with my current difficulties and problems by – actively tackling tasks.” The scale has displayed good internal consistency (α = .88), evidence for convergent and divergent validity as well as evidence of change sensitivity in the course of psychotherapy (Schürmann-Vengels et al., 2022; Victor et al., 2019).

##### Resilience of Patients

Connor-Davidson Resilience Scale (CD-RISC; 10 Item version, German adaption by Sarubin et al., 2015). As described, psychological resilience is a dynamic and multidimensional trait that enables a successful adaptation to adversities and stressors (Connor & Davidson, 2003). The CD-RISC is an internationally used self-report (range 1-7) to access general resilience. Item example: “I am able to adapt when some things change”. The German adaptation with 10 items has shown good internal consistency (α = .84), good retest-reliability (*rtt* = .81, *p* < .001) and evidence for convergent validity (Sarubin et al., 2015).

##### General Self-Esteem

Rosenberg Self-Esteem Scale (RSES; von Collani & Herzberg, 2003). As described, self-esteem is defined as the degree, a person positively consider his/her characteristics or abilities (Brown, 2007). The RSES is an internationally used self-report with 10 items (range 0-3) to assess a sum score of general self-esteem. Item example: “I am able to do things as well as most other people.” The German version shows good internal consistency (α = .83-.88) as well as evidence of criterion and construct validity (von Collani & Herzberg, 2003).

#### Intervention Analysis – Secondary Outcome

##### General Psychopathology

Brief Symptom Inventory (BSI; Franke, 2000). The BSI is an internationally used self-report with 53 items (range 0-4). The BSI was chosen as an outcome measure of the intervention analysis to analyze the effects between conditions on symptom improvement. We decided to include this version in addition to the economical process measure of psychopathology (SCL-K-9), because its subscales delivers specific information on the improvement of different mental disorders and it is more comparable to outcome measures in other intervention studies. Item example: “During the last past seven days, how much were you distressed by: feeling lonely.” The German version has shown excellent psychometric properties in clinical samples and is one of the most used instruments in psychotherapy research (Geisheim et al., 2002).

##### Working Alliance

WAI-SR (Wilmers et al., 2008). Description of the instrument, see process analysis – further measures. We further included the WAI-SR as a secondary outcome to analyze whether the conditions have specific influence on general alliance.

#### Further Measures

##### Self-Efficacy

General Self-Efficacy Scale (GES; Schwarzer & Jerusalem, 1999). Self-efficacy refers to the subjective belief that a person is confident that his/her actions lead to successful/targeted outcomes (Bandura, 1977). Several studies have suggested the beneficial effects of self-efficacy on mental health (e.g. Schönfeld et al., 2016). The GES was, therefore, included as a possible predictor of the slope of PA and NA in the process analysis. The GES is an internationally used self-report with ten items (range 0-3). Item example: “I can always manage to solve difficult problems if I try hard enough.” The instrument has shown excellent internal consistency (α = .80-.90) as well as good predictive quality in psychotherapeutic contexts (Schwarzer & Jerusalem, 1999).

##### General Mental Imagery Ability

Spontaneous Use of Imagery Scale (SUIS; German adaptation by Görgen et al., 2016). The SUIS is an internationally used self-report. The German adaptation consists of 17 items (range 1-5) and showed good internal consistency (α = .85), evidence for convergent validity as well as high correlations to the original scale (Görgen et al., 2016). Item example: “When I think about visiting a relative, I almost always have a clear mental picture of him/her.”

#### Observer Rating

To assess relevant aspects on a minute-by-minute basis within treatment sessions, the Resource-oriented Microprocess Analysis will be applied (ROMA; Flückiger & Grosse Holtforth, 2008). The instrument is a coding system of different aspects of resource activation (personal resources, motivational resources, reframing of problems, global resource activation) and PA from video recordings of treatment sessions. The coding system has shown good to excellent interrater reliability for patients and therapists (Κ = .82 - .99).

Independent research assistants will analyze the videotapes. The specific application of the observer rating will be applied in a prior workshop conducted by CF.

### Statistical Analysis

For the main hypotheses, measures will display a nested data structure (sessions at level 1 are nested with patients at level 2, nested with therapists at level 3). Therefore, we will use MLM as recommended by Baldwin et al. (2014). Separate MLM analyses will be conducted for session questionnaires of the process analysis with twelve measurements, and change questionnaires of the intervention analysis with four measurements. Time will be a within-subject factor and treatment condition a between-subject factor in both procedures. Main effects and time*condition interactions will be analyzed. We hypothesize that the slope in the PMI will increase significantly stronger compared to the NMI and TAU conditions. Possible level-2-predictors, especially for the slope of PA, will be considered. Both per-protocol and intention-to-treat analyses will be conducted.

## Discussion

The effects of PA on broadening attention and flexibility, as well as building resources and mental health, are well researched. Various findings showed a down-regulation of PA in persons with mental disorders. Despite its relevance for psychotherapy patients, there is a dearth of knowledge about the course and systematic implementation of PA in CBT. No study so far has attempted to activate PA at the start of CBT sessions to explore possible effects on process and outcome. To fill this research gap, we developed the *PACIfIC*-study.

### Innovations

The present study includes various innovative aspects: first, in line with other studies (e.g., Mander et al., 2019; Flückiger et al., 2018) a new perspective of standardized strategies to introduce psychotherapy sessions is taken up. Second, our study will have an explicit focus on PA and its impact on CBT. Third, to the best of our knowledge, this study is the first one implementing an economical in-session intervention to enhance PA in psychotherapeutic treatment. Forth, this implementation trial uses a cross-therapist design to systematize on therapist effects (e.g., Flückiger et al., 2018; Schiefele et al., 2017).

### Bias Minimization

An independent research assistant will randomize patients to treatment arms. Patients, therapists and researchers involved in the data collection and evaluation will be blind to the randomization. In addition, patients will be blind to the specific hypotheses. Patient characteristics will be compared between conditions to check for possible confounding variables. A cross-therapist design is applied to minimize therapist effects. The application of standardized audiotape records will additionally reduce possible therapists effects. The MLM will decrease an overestimation of effects emerging from the nested data structure and is robust in handling possible missing data.

### Adherence Strategies

All study coworkers will use standardized materials to enhance adherence. Patients in both active conditions will be trained in the respective mental imagery intervention during the diagnostic phase. Patients will get email address and phone number of the study coworker who conducted the training to be reachable if any problems or questions occur. Each therapist involved in the study will be informed about study procedures and technical handling. Further, a list with the most important study aspects will be handed out to therapists before enrollment. This list will also be placed in every therapist-office. A study coworker will regularly contact each therapist in person to enhance study compliance. Furthermore, data collection will regularly be checked to discuss irregularities.

### Identification of Risks

Previous studies indicated that potential risks of the mental imagery interventions are low (Blackwell et al., 2015; Dainer-Best et al., 2018; Ji et al., 2017; Renner et al., 2017). However, possible risks of the interventions lie in the intensification of specific symptoms (psychopathology) or emotional states. Affect and psychopathology measures in the process analysis will be used for a post-hoc check of unwanted effects/trajectories of involved patients. Furthermore, possible negative effects will be documented from patients’ (conclusion survey) and therapists’ perspective (regularly exchange with study coworkers).

### Conclusion

Our study will examine patients’ PA in an early phase of CBT treatment. It will further test a brief mental imagery intervention to foster PA in an outpatient sample. Our results may identify PA as a complementary factor in psychopathology and how it is affected by psychotherapy. Our results could furthermore implement the idea for strengths-based interventions as a transdiagnostic strategy to improve treatment outcome.

## Supplementary Materials

The Supplementary Materials contain standardized scripts of the positive mental imagery induction (PMI) and neutral mental imagery induction (NMI):

- Appendix A – Positive mental imagery induction (PMI)
- Appendix B – Neutral mental imagery induction (NMI)

For access see Index of Supplementary Materials below.

10.23668/psycharchives.6977 Supplement 1 Supplementary materials to "A mental imagery micro-intervention to increase positive affect in outpatient CBT sessions (PACIfIC): Study protocol of a randomized controlled implementation trial"

Schürmann-VengelsJ.
VictorP. P.
OdyniecP.
FlückigerC.
TeismannT.
WillutzkiU.
 (2022). Supplementary materials to "A
mental imagery micro-intervention to increase positive affect in
outpatient CBT sessions (PACIfIC): Study protocol of a randomized
controlled implementation trial"
[Additional information]. PsychOpen. 10.23668/psycharchives.6977
PMC966742436397941

## Trial registration

Trial registered at ClinicalTrials.gov, Identifier: NCT03767101 (registered December 6, 2018), https://clinicaltrials.gov/ct2/show/NCT03767101
