# Supplementary material for: A Mental Imagery Micro-Intervention to Increase Positive Affect in Outpatient CBT Sessions (PACIfIC): Study Protocol of a Randomized Controlled Implementation Trial
Source: Clin Psychol Eur. 2022 Jun 30;4(2):e7043. doi: 10.32872/cpe.7043 (PMC9667424; doi:10.32872/cpe.7043)
Supplement: Supplement 1 [file cpe-04-7043-s01.pdf]

Supplement of the following paper:

Schürmann-Vengels, J., Victor, P.P., Odyniec, P., Flückiger, C., Teismann, T., & Willutzki, U. A mental imagery micro-intervention to increase positive affect in outpatient CBT sessions (PACIfIC): study protocol of a randomized controlled implementation trial. *Clinical Psychology in Europe*.

Appendix A – Positive mental imagery induction (PMI)

Please sit down on your chair and make yourself very comfortable. Stay completely with yourself. As discussed, you do not have to speak aloud during the exercise. Linger in your imagination. You may want to close your eyes or focus on a specific point in the room. Slowly calm down and take a deep breath. You are here because at least some things in your life are supposed to change - before you get into the session right there, I'd like to invite you to take a look at things in your life that can stay as they are now. Please focus your attention to pleasant, good, maybe even beautiful moments and events of the last week ... Now choose an event or a moment that was pleasant, good or even happy for you ... If you come up with several things, please choose one of them today ... If nothing comes to your mind in the moment, just go back in time ... Imagine the moment in your mind's eye. As if you are back in the moment and see it with your own eyes. The details and what happened. Take a close look. Imagine the moment so that you could describe it to someone who was not there. – 30 sec pause –
Please stay with your picture. Now I will give you some suggestions for deepening. Just stay with yourself and imagine the moment in detail: take a look around, what do you see.
See if you find something special. Listen carefully, to the sounds in the moment. Maybe you notice a certain smell. Look what you are doing. And how you feel about yourself. Take notice of your body. Is it warm or cool? Take in your breath. And take some time, stay a while in this moment and look around – 20 sec pause – Capture this image and the feeling, make a kind of inner snapshot for yourself. And, as it suits you, come back to the therapy room. Maybe breathe in and out a few more times. Feel the contact with the floor or armchair. Maybe you like to stretch or move something? Finally, take a minute and tell your therapist about the impressions and your ideas before the two of you come together to today's topics.

Duration: 6.27 min

Appendix B – Neutral mental imagery induction (NMI)

Please sit down on your chair and make yourself very comfortable. Stay completely with yourself. As discussed, you do not have to speak aloud during the exercise. Linger in your imagination. You may want to close your eyes or focus on a specific point in the room. Slowly calm down and take a deep breath. Before you get into the session right there, I'd like to invite you to take a look at the last week. Please focus your attention to everyday events of the last week ... Now choose an event or a moment that that was neither particularly positive nor particularly negative, did not trigger any strong feelings in you... If you come up with several things, please choose one of them today ... If nothing comes to your mind in the moment, just go back in time ... Imagine the moment in your mind's eye. As if you are back in the moment and see it with your own eyes. The details and what happened. Take a close look. Imagine the moment so that you could describe it to someone who was not there. – 30 sec pause –

Please stay with your picture. Now I will give you some suggestions for deepening. Just stay with yourself and imagine the moment in detail: take a look around, what do you see.

See if you find something special. Listen carefully, to the sounds in the moment. Maybe you notice a certain smell. Look what you are doing. And how you feel about yourself. Take notice of your body. Is it warm or cool? Take in your breath. And take some time, stay a while in this moment and look around – 20 sec pause – Capture this image and the feeling, make a kind of inner snapshot for yourself. And, as it suits you, come back to the therapy room. Maybe breathe in and out a few more times. Feel the contact with the floor or armchair. Maybe you like to stretch or move something? Finally, take a minute and tell your therapist about the impressions and your ideas before the two of you come together to today's topics.

Duration: 6.09 min
